# Glymphatic-related imaging findings in type 2 diabetes mellitus: a systematic review and exploratory meta-analysis of DTI-ALPS studies

**DOI:** 10.3389/fnagi.2026.1818446

**Published:** 2026-05-21

**Authors:** Yaxi Chen, Junhuai Zhang, Xiaoyu Zheng, Yuan Zhou, Xiaofeng Hou

**Affiliations:** 1 School of Clinical Medicine, Chongqing Medical and Pharmaceutical College, Chongqing, China; 2 Holistic Center for Neurological Diseases, The People’s Hospital of Chongqing Yubei District, Chongqing, China

**Keywords:** cognitive impairment, DTI-ALPS, glymphatic system, meta-analysis, perivascular diffusion, type 2 diabetes mellitus

## Abstract

**Background:**

Type 2 diabetes mellitus (T2DM) is associated with white matter microstructural, neurovascular, and cognitive changes. Perivascular fluid dynamics may also be altered in T2DM. The diffusion tensor image analysis along the perivascular space (DTI-ALPS) index is an indirect imaging measure of perivascular water diffusivity, but its behavior across different clinical phenotypes of T2DM has not been quantitatively synthesized.

**Methods:**

We conducted a systematic review and meta-analysis of DTI-ALPS studies comparing individuals with T2DM and healthy controls. Random-effects models with Hartung–Knapp–Sidik–Jonkman (HKSJ) adjustment were applied. Subgroup analyses were performed according to clinical phenotype, distinguishing uncomplicated T2DM from T2DM with established complications.

**Results:**

Seven studies comprising 532 participants met the inclusion criteria. In the uncomplicated T2DM subgroup (*k* = 4), the pooled Hedges’ *g* was −0.89 [95% CI (−1.45, −0.34), HKSJ-adjusted; *p* = 0.015; *I*^2^ = 57.9%], reaching statistical significance. In the primary analysis of complicated T2DM (*k* = 3), the pooled Hedges’ *g* was −1.34 [95% CI (−3.55, +0.86), HKSJ-adjusted; *p* = 0.119; *I*^2^ = 90.7%], and did not reach statistical significance. The formal between-subgroup test was not significant in the primary analysis (*Q*_between_ = 0.66, df = 1, *p* = 0.416). A *post-hoc* sensitivity analysis of the complicated subgroup including Hu 2025 (*k* = 4) yielded a pooled Hedges’ *g* of −1.73 [95% CI (−3.42, −0.04); *p* = 0.047], whereas the between-subgroup difference remained non-significant (*Q*_between_ = 2.16, df = 1, *p* = 0.141). Across all seven studies and both subgroups, point estimates consistently favored lower DTI-ALPS indices in T2DM, although certainty of evidence was rated as very low for both primary outcomes.

**Conclusion:**

This meta-analysis showed a consistent trend toward lower DTI-ALPS indices in T2DM than in healthy controls. The uncomplicated subgroup reached statistical significance, whereas the complicated subgroup primary analysis did not. No significant between-subgroup difference was detected. These findings should be interpreted cautiously given the limited number of studies, substantial heterogeneity in the complicated subgroup, and the indirect nature of the DTI-ALPS metric.

**Systematic review registration:**

https://www.crd.york.ac.uk/PROSPERO/view/CRD420261303719, PROSPERO CRD420261303719.

## Introduction

Type 2 diabetes mellitus (T2DM) is increasingly recognized as a condition associated not only with systemic metabolic dysfunction but also with structural and functional brain abnormalities. Neuroimaging studies have reported cerebral atrophy, cerebral small-vessel disease, and white matter microstructural alterations in individuals with T2DM (Biessels and Reijmer, 2014). T2DM is also associated with an increased risk of cognitive decline and dementia, with population-based studies indicating a 40–60% increased risk compared with non-diabetic populations (Vagelatos and Eslick, 2013; Biessels and Whitmer, 2020; Srikanth et al., 2020). Diabetes-related cognitive impairment is clinically relevant because it can adversely affect self-management capacity and increase vulnerability to adverse outcomes. Despite these observations, the mechanisms linking chronic metabolic dysregulation to brain vulnerability remain incompletely understood (Biessels et al., 2008).

Recent interest has extended to cerebral perivascular fluid dynamics as a potential contributor to diabetes-related brain abnormalities. The glymphatic pathway is a proposed perivascular fluid-transport system that may contribute to interstitial solute clearance in the brain (Iliff et al., 2012; Jessen et al., 2015; Mestre et al., 2018). Diabetes-related metabolic and vascular disturbances could plausibly affect this pathway, but glymphatic function cannot be measured directly *in vivo* in humans. Diffusion tensor image analysis along the perivascular space (DTI-ALPS) has therefore been proposed as an indirect, non-invasive imaging proxy for perivascular diffusion behavior (Taoka et al., 2017).

An increasing number of neuroimaging studies have applied the DTI-ALPS index in T2DM cohorts, but the reported findings remain heterogeneous. Some studies have reported lower DTI-ALPS values in T2DM cohorts with complications (Wang et al., 2025; Yu B. et al., 2024), whereas others have found more modest differences in uncomplicated cohorts (Diao et al., 2025), and imaging methods have varied substantially across reports. Accordingly, we conducted an exploratory systematic review and meta-analysis to synthesize the available evidence on DTI-ALPS findings in T2DM. Specifically, we aimed to: (1) characterize the direction and magnitude of DTI-ALPS differences between individuals with T2DM and healthy controls; (2) examine whether findings differ between uncomplicated and complicated clinical subgroups; and (3) qualitatively assess important sources of methodological heterogeneity across studies. Given the limited number of eligible studies and the indirect nature of DTI-ALPS, this synthesis is intended to be hypothesis-generating rather than confirmatory.

## Methods

### Study registration and protocol

The study protocol, including the research question, search strategy, and eligibility criteria, was established *a priori* and initiated on January 7, 2026. The protocol was prospectively registered with the International Prospective Register of Systematic Reviews (PROSPERO; registration number CRD420261303719). Reporting follows the PRISMA 2020 guidelines (Page et al., 2021). Because the number of eligible studies was anticipated to be small and the between-study variability in cohorts and imaging protocols was expected to be substantial, the synthesis was pre-specified as an exploratory meta-analysis rather than as a confirmatory analysis. This framing is reflected in the title, abstract, and interpretive language throughout the manuscript.

### Literature search and selection

A systematic search was conducted across PubMed, Embase, and Web of Science to identify studies investigating glymphatic-related imaging alterations in T2DM using the DTI-ALPS index. The pre-specified search window extended up to February 2026. The detailed search strings for all databases are provided in Supplementary Table 1. Titles and abstracts were independently screened by two reviewers (Y.C. and J.Z.), and disagreements were resolved through discussion with a third reviewer (X.H.). Full-text assessment of potentially eligible records was performed independently by the same two reviewers, with disagreements again resolved by consensus with the third reviewer.

Studies were included if they: (1) enrolled adults diagnosed with T2DM and a healthy control group; (2) reported the DTI-ALPS index as a primary or secondary outcome; and (3) provided sufficient summary statistics (mean and standard deviation, or median and interquartile range) to permit effect size calculation.

Studies were excluded if they were: (1) case reports, conference abstracts, reviews, editorials, or methodological papers without original primary data; (2) based on overlapping or duplicated patient cohorts with another included study; or (3) enrolled participants with major neurological comorbidities, including recent cerebrovascular events (stroke within the preceding 6 months), intracranial tumors, or traumatic brain injury, because these conditions are known to substantially alter diffusion metrics (Alexander et al., 2007).

Three studies identified through the systematic search were not included in the primary quantitative synthesis: Yang 2020 (Yang et al., 2020) was excluded because the original publication presented DTI-ALPS values only as scatter plots without summary markers, precluding reliable effect-size extraction; Xu 2026 (Xu et al., 2026) was excluded because it did not report sufficient quantitative data to permit valid effect-size calculation; and Hu 2025 (Hu et al., 2025) was evaluated as a *post-hoc* sensitivity analysis rather than included in the primary synthesis, for reasons explained in the *post-hoc* sensitivity analysis subsection below. Detailed exclusion rationales for Yang 2020 and Xu 2026 are provided in Supplementary Methods Section S4.

### Quality assessment

Methodological quality was assessed using the Newcastle–Ottawa Scale (NOS). Two reviewers independently evaluated each study across three domains (selection, comparability, and outcome), and discrepancies were resolved through discussion with a third reviewer. NOS scores for the included studies ranged from 7 to 8, indicating overall moderate to high methodological quality. Detailed quality assessments are presented in Table 1 and Supplementary Table 2.

**Table 1:** Characteristics of the included studies evaluating the DTI-ALPS index in Type 2 diabetes mellitus.

Study ID	N (T / HC)	Age (y), T / HC	Male, n (%), T / HC	BMI (kg/m^2^), T / HC	HbA1c (%), T / HC	Cognitive score, T / HC	Scale	MRI (Field / b / dir)	Software	Voxel size (mm^3^)	NOS
Uncomplicated T2DM											
Tian et al. (2024)	22/22	49.4 ± 7.4/51.3 ± 6.8	12 (54.5) / 11 (50.0)	25.75 ± 3.83/23.38 ± 2.11	8.51 ± 3.42/5.64 ± 0.42	29.00 ± 1.38/29.77 ± 0.53	MMSE	3.0 T / 1,000/99	FSL	2.0 × 2.0 × 2.0	7
Tuerxun et al. (2024)	18/30	72.6 ± 6.0/70.3 ± 4.8	13 (72.2) / 14 (46.7)	24.10 ± 2.90/21.60 ± 3.00	6.60 ± 0.90/5.50 ± 0.30	25.80 ± 2.60/26.20 ± 2.50	MoCA	3.0 T / 1,000/64	FSL	1.8 × 1.8 × 1.8	7
Yu S. C. et al. (2024)	41/27	60.5 ± 8.9/58.0 ± 7.6	21 (51.2) / 10 (37.0)	25.48 ± 3.09/23.60 ± 3.22	7.58 ± 1.45/5.50 ± 0.26	25.42 ± 2.37/26.33 ± 2.57	MoCA	3.0 T / 1,000/64	FSL	2.0 × 2.0 × 2.0	7
Roy et al. (2026)	78/106	56.5 ± 7.5/54.7 ± 6.5	39 (50.0) / 53 (50.0)	29.45 ± 5.00/26.28 ± 4.20	7.04 ± 1.30/5.30 ± 0.40	25.90 ± 2.50/27.10 ± 2.30	MoCA	3.0 T / 800/30	DTI Studio	1.8 × 1.8 × 1.7	7
T2DM with complications											
Diao et al. (2025) ^a^	39/37	53.50 ± 8.08/51.67 ± 10.80	19 (48.7) / 11 (29.7)	23.40 ± 4.02/22.79 ± 2.17	7.65 ± 2.12/5.70 ± 0.31	23.00 ± 3.08/27.67 ± 0.77	MoCA	3.0 T / 1,000/99	FSL / MATLAB	2.0 × 2.0 × 2.0	7
Wang et al. (2025)	40/40	58.65 ± 6.44/59.38 ± 6.50	21 (52.5) / 18 (45.0)	24.96 ± 3.33/24.20 ± 1.53	10.06 ± 2.10/5.00 ± 0.63	17.45 ± 3.00/26.75 ± 0.84	MoCA	3.0 T / 1,000/64	DTI Studio	3.0 × 2.0 × 2.0	8
Yu B. et al. (2024)	25/25	55.5 ± 10.7/54.5 ± 5.3	19 (76.0) / 15 (60.0)	NR / NR	6.9 ± 1.6 / NR	23.2 ± 4.4 / NR	MoCA	3.0 T / 1,000/64	FSL / MRtrix3	NR	7
Additional study included in sensitivity analysis only											
Hu et al. (2025) ^b^	35/35	68.20 ± 9.15/64.03 ± 11.26	23 (65.7) / 21 (60.0)	23.91 ± 2.29/24.93 ± 2.22	7.16 ± 1.49/5.62 ± 0.30	21.83 ± 1.76/27.80 ± 1.18	MoCA	3.0 T / 1,000/64	DSI Studio	1.8 × 1.8 × 2.0	8

T, type 2 diabetes mellitus group; HC, healthy control group; BMI, body mass index; HbA1c, glycated hemoglobin; MoCA, Montreal Cognitive Assessment; MMSE, Mini-Mental State Examination; NR, not reported; FSL, FMRIB Software Library; DTI Studio, Diffusion Tensor Imaging Studio; DSI Studio, Diffusion Spectrum Imaging Studio; NOS, Newcastle–Ottawa Scale; T2DM, type 2 diabetes mellitus.

Data presentation: Continuous variables are expressed as mean ± standard deviation (SD). When the original publications reported values as median and interquartile range, summary statistics were converted to mean ± SD using the formulas of Wan et al. (2014).

Subgroup classification. Uncomplicated T2DM refers to cohorts of patients without documented major diabetic complications and with preserved cognitive function at the cohort level. T2DM with complications refers to cohorts of patients with documented diabetic complications, including cognitive impairment or diabetic kidney disease.

MRI parameters: The MRI column is presented as magnetic field strength (Tesla)/*b*-value (s/mm^2^)/number of diffusion-encoding directions. All included studies used 3.0 Tesla scanners and single-shot echo-planar diffusion-weighted imaging.

Quality assessment: Study quality was evaluated using the Newcastle–Ottawa Scale (NOS); scores ≥ 7 indicate moderate to high methodological quality.

a For this multi-arm study (HC, *n* = 37; T2DM without MCI, *n* = 37; T2DM with MCI, *n* = 39), the T2DM-MCI subgroup versus the healthy control group was included in the quantitative synthesis, consistent with the pre-specified phenotypic classification for the T2DM with complications subgroup. The T2DM without MCI subgroup was not included, to avoid unit-of-analysis concerns associated with using the same control group in more than one comparison. Age, BMI, HbA1c, and MoCA values for Diao et al. (2025) were reported in the original publication as median and interquartile range and were converted to mean ± SD using the formulas of Wan et al. (2014).

b For this multi-arm study (HC, *n* = 35; T2DM without MCI, *n* = 35; T2DM with MCI, *n* = 35), the T2DM-MCI subgroup versus the healthy control group was included in the *post-hoc* sensitivity analysis of the T2DM with complications subgroup (Hu et al., 2025).

### Assessment of certainty of evidence

The certainty of evidence for each outcome was assessed using the Grading of Recommendations, Assessment, Development and Evaluations (GRADE) framework (Guyatt et al., 2008). In the GRADE approach, evidence from observational studies begins at a “low” level of certainty and may be further downgraded based on five domains: risk of bias, inconsistency, indirectness, imprecision, and publication bias. Each outcome was independently rated by two reviewers (Y.C. and J.Z.), and discrepancies were resolved through discussion with a third reviewer (X.H.). GRADE assessments were performed using GRADEpro GDT software (McMaster University and Evidence Prime, Canada). The certainty of evidence was categorized as high, moderate, low, or very low for each primary outcome.

### Data extraction

Data extraction was performed independently by two reviewers (Y.C. and J.Z.), with disagreements resolved by discussion with a third reviewer (X.H.). Extracted items included: sample sizes; mean and standard deviation of the DTI-ALPS index for each comparison group; clinical variables [age, disease duration, body mass index, HbA1c levels, cognitive scores (MoCA or MMSE)]; and key imaging acquisition parameters (field strength, b-value, number of diffusion-encoding directions, voxel size, post-processing software, and ROI placement strategy). HbA1c values are reported in NGSP units (%) as provided in the original studies; IFCC units (mmol/mol) were not consistently reported and were therefore not uniformly available for conversion.

When summary data were reported as medians and interquartile ranges rather than means and standard deviations, values were converted using the formulas of Wan et al. (2014). Specifically, for a sample of size n with reported median m, first quartile *q_1_*, and third quartile *q_3_*, the estimated mean was computed as (*q_1_* + *m* + *q_3_*)/3 and the estimated standard deviation as (*q_3_* − *q_1_*)/(2 × *Φ*^−1^((0.75n − 0.125)/(n + 0.25))), where Φ^−1^ denotes the inverse standard normal cumulative distribution function.

Three included studies required individualized data-extraction procedures because the original publications did not report tabular mean ± standard deviation values for the DTI-ALPS index in a directly extractable form: Yu B 2024 [graphical median-IQR reading from violin plots, converted via Wan et al. (2014)], Tian 2024 [mean and standard deviation reconstructed from published 95% confidence intervals using a t-distribution-based inversion in accordance with Cochrane Handbook Chapter 6.5.2.3 (Higgins et al., 2023)], and Diao 2025 (median–IQR conversion via Wan 2014, with selection of a single comparison from a three-arm design to avoid unit-of-analysis concerns). Detailed extraction procedures, source-paragraph references, and the rationale for each decision are provided in Supplementary Methods Sections S1 [Yu B 2024 (Yu B. et al., 2024)], S2 [Tian 2024 (Tian et al., 2024)], and S3 [Diao 2025 (Diao et al., 2025)]. For Tian 2024, an additional pre-specified sensitivity analysis using an alternative face-value reading of the Tian 2024 first Results paragraph is reported in Supplementary materials, and the direction of the pooled effect is preserved under both extractions.

### Subgroup classification

For quantitative synthesis, studies were categorized into two pre-specified clinical-phenotype subgroups, reflecting the cohort-level clinical profile of the T2DM group in each included study: (1) Uncomplicated T2DM: patients without documented major diabetic complications and with preserved cognitive function at the cohort level. (2) T2DM with complications: patients with documented diabetic complications, including cognitive impairment (defined by reduced performance on standardized cognitive assessments at the cohort level) or diabetic kidney disease.

We emphasize that this classification reflects cohort-level clinical phenotype, not disease stage. Cohorts classified in the two subgroups differ not only in complication status but also in correlated variables such as disease duration, HbA1c, age, and imaging acquisition parameters. We therefore describe any observed differences between these subgroups as “differences between clinical subgroups” rather than as evidence of a temporal progression. This terminology is used consistently throughout the Results and Discussion.

### MRI acquisition and DTI-ALPS calculation

All included studies acquired diffusion-weighted imaging on 3.0 Tesla scanners using single-shot echo-planar sequences and computed the DTI-ALPS index using the formula originally proposed by Taoka et al. (2017). The standard processing pipeline consisted of: (1) correction for eddy-current distortions, susceptibility-induced distortions, and head motion using FSL (Jenkinson et al., 2012) or equivalent toolboxes (Andersson and Sotiropoulos, 2016); (2) voxel-wise diffusion tensor estimation to generate diffusivity maps along the x, y, and z axes; (3) bilateral region-of-interest placement at the level of the lateral ventricle body, in areas corresponding to projection fibers (medial) and association fibers (lateral); and (4) calculation of the DTI-ALPS index as the ratio of the mean of x-axis diffusivities in the projection and association fiber regions to the mean of the perpendicular y- and z-axis diffusivities in the same regions. Lower DTI-ALPS values were interpreted as indicating reduced diffusivity along the direction of the perivascular space and are interpreted in this manuscript as an indirect imaging index of perivascular water diffusivity, not as a direct measure of glymphatic clearance.

Acquisition and post-processing parameters varied across studies in b-value, number of diffusion-encoding directions, voxel size, post-processing software, ROI placement strategy, and the reported ALPS index laterality; full per-study details are tabulated in Table 1, and a structured comparison of common elements and sources of variability is provided in Supplementary Methods Section S5. Because of the limited number of eligible studies, formal meta-regression to isolate the effect of individual methodological variables was not performed.

### Statistical analysis

Meta-analyses were performed using Stata (version 18.0; StataCorp, USA). Standardized mean differences were expressed as Hedges’ *g* with 95% confidence intervals, computed from the reported sample sizes, means, and standard deviations of the DTI-ALPS index in the T2DM and healthy control groups of each included study. Pooled estimates were obtained using a random-effects model with restricted maximum likelihood (REML) estimation of the between-study variance *τ^2^* (DerSimonian and Laird, 1986).

Given the small number of studies included in each subgroup analysis, statistical inference was based on the Hartung–Knapp–Sidik–Jonkman (HKSJ) adjustment, which provides more conservative confidence intervals under small-sample conditions (Hartung and Knapp, 2001; Sidik and Jonkman, 2002). Conventional random-effects estimates were retained for descriptive and visualization purposes, whereas HKSJ-adjusted results were used as the primary basis for statistical inference.

Between-study heterogeneity was assessed using Cochran’s *Q* test and quantified using the *I^2^* statistic (Higgins et al., 2003). Sensitivity analyses were conducted using a leave-one-out approach to evaluate the robustness of pooled estimates to the contribution of any single study. The leave-one-out estimates are reported in the Supplementary materials for all primary and sensitivity analyses. Statistical significance was set at *p* < 0.05 (two-tailed).

### Formal between-subgroup difference test

To directly test whether the two clinical-phenotype subgroups (uncomplicated T2DM and T2DM with complications) differed in their pooled DTI-ALPS effect estimates, we conducted a formal between-subgroup comparison using Cochran’s *Q*_between_ statistic, following the subgroup-summary approach recommended in the Cochrane Handbook for Systematic Reviews of Interventions (Higgins et al., 2023). The subgroup-specific random-effects pooled estimates and their standard errors were used to compute *Q*_between_, which follows a chi-squared distribution with degrees of freedom equal to the number of subgroups minus one. Statistical significance was evaluated at the conventional *α* = 0.05 threshold. The test was applied both to the primary synthesis and to the sensitivity analysis described below.

### *Post-hoc* sensitivity analysis

To assess the robustness of the complicated T2DM subgroup pooled estimate to between-study variability in reported precision, we conducted a *post-hoc* sensitivity analysis incorporating Hu 2025. The control group of Hu 2025 exhibited a substantially smaller standard deviation of the DTI-ALPS index (SD = 0.055) than the control groups of the other included studies, reflecting a more homogeneous control cohort and correspondingly higher reported precision. Because inverse-variance weighting in random-effects meta-analysis is sensitive to reported study precision, we assessed whether the inclusion of Hu 2025 in the quantitative synthesis would alter the direction, magnitude, or statistical stability of the complicated-subgroup pooled estimate obtained from the studies whose control-group precision was more comparable.

For the sensitivity analysis, we used the T2DM-MCI subgroup of Hu 2025 (*n* = 35) versus the healthy control group (*n* = 35), consistent with our pre-specified phenotypic classification for the complicated T2DM subgroup. The T2DM without MCI subgroup of Hu 2025 was not included in the sensitivity analysis, for the same unit-of-analysis reasons that applied to the multi-arm handling of Diao 2025 (see Supplementary Methods Section S3). The sensitivity analysis was evaluated on three criteria: (i) whether the direction of effect was preserved; (ii) whether HKSJ-adjusted statistical inference changed qualitatively relative to the primary analysis; and (iii) whether leave-one-out stability of the pooled estimate was affected by inclusion of the additional study. The formal between-subgroup difference test described above was applied to both the primary synthesis and the sensitivity analysis.

## Results

### Study selection and participant characteristics

**Figure 1:**
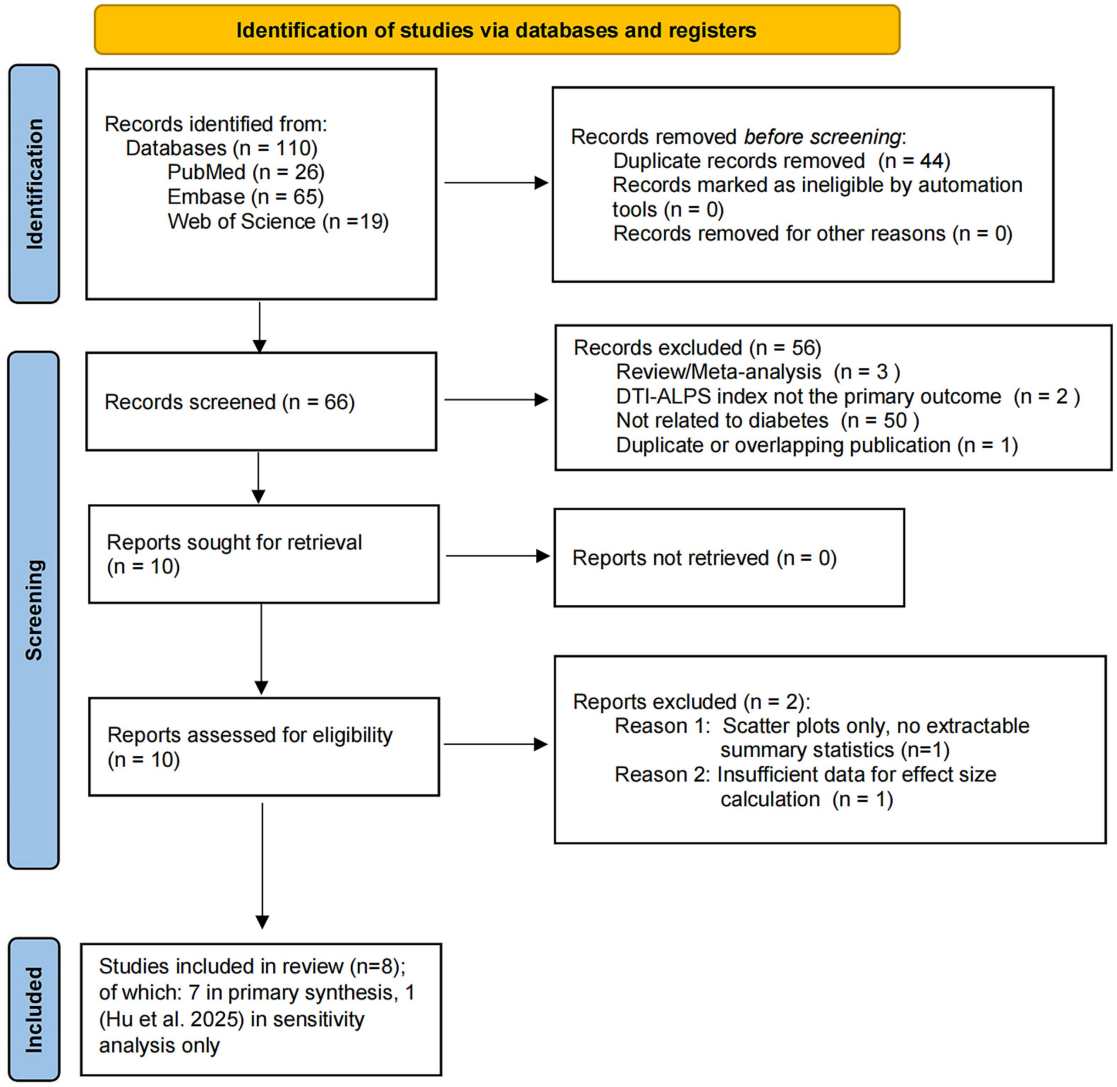
PRISMA 2020 flow diagram of the study selection process. The flowchart illustrates the systematic identification, screening, and inclusion of studies for this meta-analysis. A total of 110 records were identified from electronic databases, including PubMed (*n* = 26), Embase (*n* = 65), and Web of Science (*n* = 19). After removal of duplicates (*n* = 44), 66 records underwent title and abstract screening, of which 56 were excluded for not meeting the predefined inclusion criteria. Ten full-text articles were assessed for eligibility. Two studies were excluded from the quantitative synthesis: Yang et al. (2020) because DTI-ALPS values were presented only as scatter plots without extractable summary statistics, and Xu et al. (2026) because of insufficient quantitative data for effect size calculation. Seven studies were included in the primary quantitative synthesis (*n* = 532 participants). An additional study (Hu et al., 2025) was incorporated into the *post-hoc* sensitivity analysis of the complicated T2DM subgroup.

A total of 110 records were initially identified through the systematic search (PubMed, *n* = 26; Embase, *n* = 65; Web of Science, *n* = 19). After removal of duplicates, 66 records underwent title and abstract screening, of which 56 were excluded for not meeting the predefined inclusion criteria. Ten full-text articles were assessed for eligibility, and two were excluded. Yang 2020 (Yang et al., 2020) was excluded because the original publication presented DTI-ALPS values only as scatter plots without accompanying summary statistics, which precluded the extraction of effect sizes required for quantitative synthesis. Xu 2026 (Xu et al., 2026) was excluded because it did not report sufficient quantitative data to permit valid effect size calculation. Ultimately, seven studies were included in the primary quantitative synthesis (Wang et al., 2025; Diao et al., 2025; Roy et al., 2026; Tian et al., 2024; Tuerxun et al., 2024; Yu S. C. et al., 2024; Yu B. et al., 2024), with an eighth study Hu 2025 (Hu et al., 2025) contributing to the sensitivity analysis of the complicated T2DM subgroup. The primary synthesis encompassed 532 participants (263 with T2DM and 269 healthy controls); the sensitivity analysis encompassed 602 participants. The study selection process is summarized in the PRISMA 2020 flow diagram (Figure 1).

As summarized in Table 1, the included studies collectively cover a clinical spectrum of T2DM ranging from early-stage metabolic dysfunction to advanced neurocognitive deterioration. HbA1c levels in the T2DM groups spanned a wide gradient, from relatively well-controlled levels (6.60 ± 0.90% in Tuerxun 2024) to pronounced hyperglycemia (10.06 ± 2.10% in Wang 2025). Cognitive profiles also varied across the cohorts: some studies enrolled cognitively preserved patients (Tian 2024, MMSE 29.00 ± 1.38), whereas others focused on patients with manifest cognitive impairment (Wang 2025, MoCA 17.45 ± 3.00). These cohort-level differences underlie the two pre-specified clinical-phenotype subgroups used in the quantitative synthesis.

Methodologically, all included studies used 3.0 Tesla scanners with single-shot echo-planar diffusion-weighted imaging and calculated the DTI-ALPS index using the formula originally proposed by Taoka et al. (2017). Acquisition and processing parameters varied across studies, with b-values ranging from 800 s/mm^2^ (Roy 2026) to 1,000 s/mm^2^ (all other included studies), diffusion-encoding directions ranging from 30 to 99, voxel sizes ranging from 1.7 × 1.7 × 1.7 mm^3^ to 3.0 × 2.0 × 2.0 mm^3^, and post-processing performed with either FSL (five studies) or DTI Studio (two studies). These sources of methodological variability are tabulated in Table 1 and discussed as plausible contributors to between-study heterogeneity.

As specified in the Methods, Diao 2025 contributed a single comparison to the quantitative synthesis (T2DM-MCI subgroup, *n* = 39, versus healthy controls, *n* = 37; mean ± SD = 1.60 ± 0.20 and 1.68 ± 0.23 respectively). Newcastle–Ottawa Scale (NOS) scores for all included studies ranged from 7 to 8, indicating moderate to high methodological quality and a low to moderate risk of bias across the evidence base.

### Primary analysis: uncomplicated T2DM

**Figure 2:**
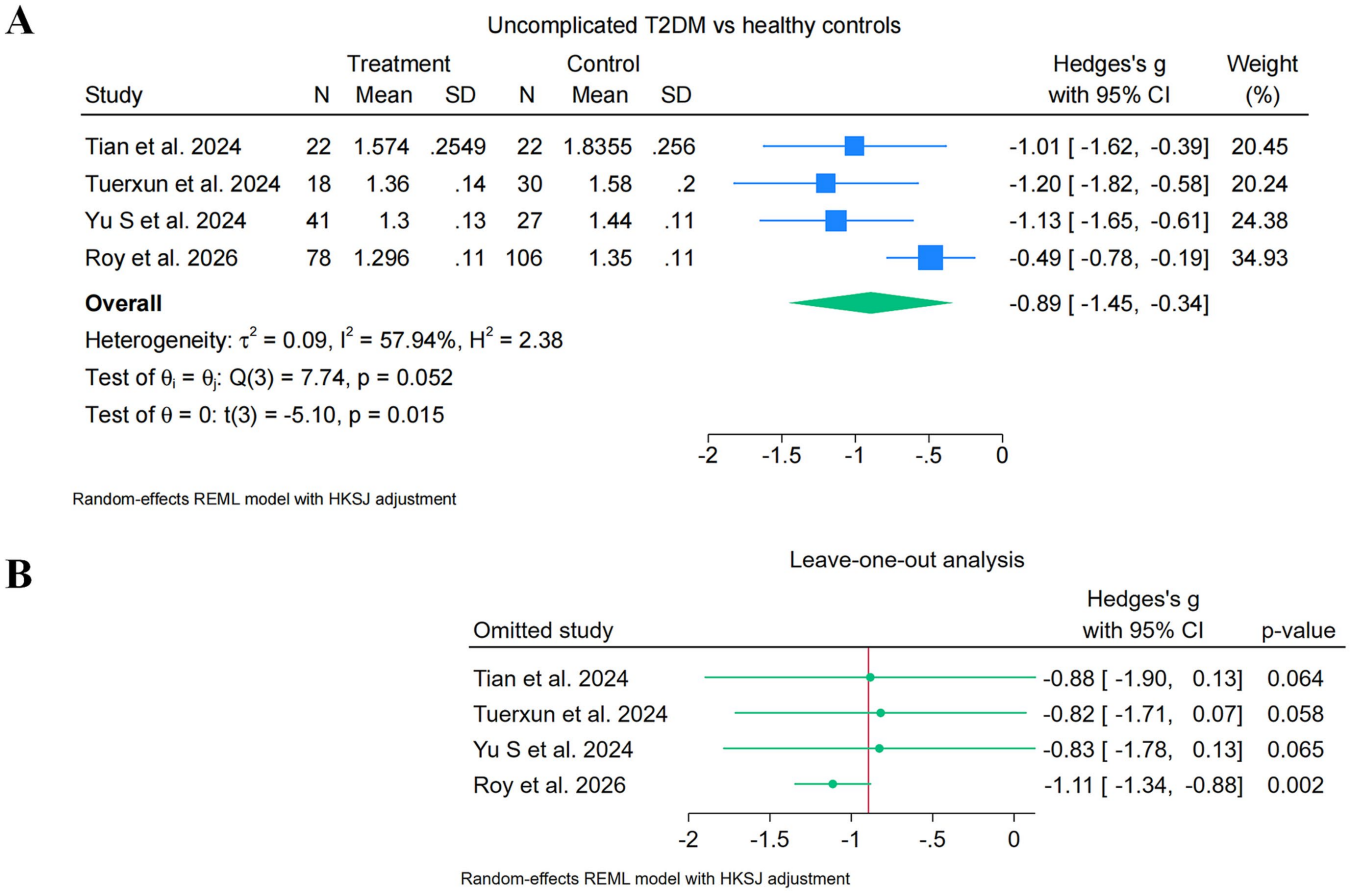
Forest plots of DTI-ALPS in uncomplicated T2DM. Random-effects meta-analysis of the DTI-ALPS index in uncomplicated type 2 diabetes mellitus (T2DM) versus healthy controls. **(A)** Primary forest plot of the four studies contributing to the uncomplicated T2DM subgroup. Individual study effect sizes are expressed as Hedges’ *g* with 95% confidence intervals. The pooled estimate was obtained using a restricted maximum likelihood (REML) random-effects model with Hartung–Knapp–Sidik–Jonkman (HKSJ) adjustment, as pre-specified in Methods. The pooled Hedges’ *g* was −0.89 (95% HKSJ-adjusted CI [−1.45, −0.34]; *t*(3) = −5.10; *p* = 0.015), with moderate to substantial between-study heterogeneity (*Q* = 7.74, *df* = 3, *p* = 0.052; *I*^2^ = 57.9%; τ^2^ = 0.090). **(B)** Leave-one-out analysis showing pooled Hedges’ *g* and 95% HKSJ-adjusted confidence intervals after sequential exclusion of each individual study. Pooled estimates across the four leave-one-out iterations ranged from −0.82 to −1.11, and the direction of effect was preserved throughout. Negative Hedges’ *g* values indicate lower DTI-ALPS index values in T2DM relative to healthy controls.

Four studies (Tian 2024; Tuerxun 2024; Yu S 2024; Roy 2026) contributed to the primary analysis of the uncomplicated T2DM subgroup, encompassing 159 patients with T2DM and 185 healthy controls. Study-level Hedges’ *g* values were: Tian 2024, *g* = −1.01; Tuerxun 2024, *g* = −1.20; Yu S 2024, *g* = −1.13; Roy 2026, *g* = −0.49. All four point estimates were in the direction of lower DTI-ALPS indices in the uncomplicated T2DM groups relative to healthy controls (Figure 2A).

Under the pre-specified restricted maximum likelihood (REML) random-effects model with Hartung–Knapp–Sidik–Jonkman (HKSJ) adjustment, the pooled Hedges’ *g* was −0.89 [95% CI (−1.45, −0.34), HKSJ-adjusted; *t*(3) = −5.10; *p* = 0.015]. Between-study heterogeneity was moderate to substantial (Cochran’s *Q* = 7.74, df = 3, *p* = 0.052; *I^2^* = 57.9%; *τ^2^* = 0.090). The pooled estimate reached statistical significance under HKSJ-adjusted inference, the direction of effect was consistent across all four contributing studies, and the upper bound of the confidence interval excluded the null. Leave-one-out sensitivity analysis (Figure 2B) showed that the overall direction of effect was not driven by any single study.

Leave-one-out pooled Hedges’ *g* values ranged from −0.82 (excluding Tuerxun 2024) to −1.11 (excluding Roy 2026), and no leave-one-out iteration produced a reversal in the direction of effect. Excluding Roy 2026, which contributed the largest sample size and the smallest single-study effect estimate, produced the most negative leave-one-out pooled estimate [*g* = −1.11, 95% CI (−1.34, −0.88); *p* = 0.002], while excluding any of the three remaining studies preserved both the direction and the approximate magnitude of the pooled estimate.

An alternative extraction of Tian 2024, using a face-value reading of the first Results paragraph of the original publication (T2DM mean = 1.7905, SD = 0.2673, *n* = 22; healthy control mean = 1.8355, SD = 0.2560, *n* = 22), was pre-specified as a sensitivity analysis. Under this alternative extraction, the uncomplicated subgroup pooled Hedges’ *g* was −0.73 [95% HKSJ-adjusted CI (−1.50, +0.05); *p* = 0.06], preserving the direction of effect but not reaching conventional statistical significance under HKSJ-adjusted inference. The leave-one-out range under the alternative extraction was −0.60 to −0.88. Detailed rationale for the primary extraction, the source-paragraph cross-validation procedure, and the full results of the alternative-extraction sensitivity analysis are provided in Supplementary Methods Sections S2, S6.

### Primary analysis: T2DM with complications

**Figure 3:**
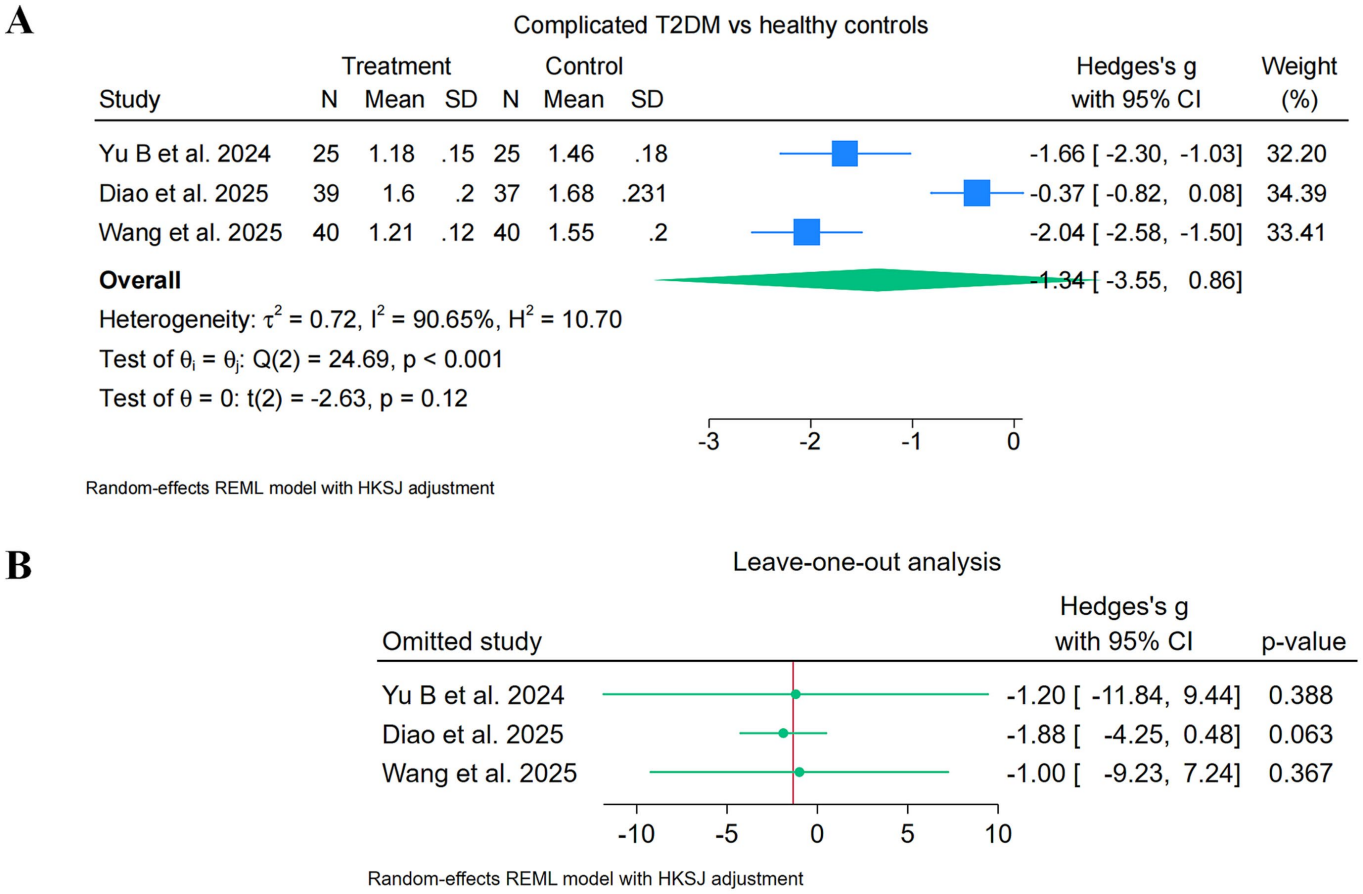
Forest plots of DTI-ALPS in complicated T2DM (primary analysis). Random-effects meta-analysis of the DTI-ALPS index in type 2 diabetes mellitus (T2DM) with complications versus healthy controls (primary analysis, *k* = 3). **(A)** Primary forest plot of the three studies contributing to the complicated T2DM subgroup. Individual study effect sizes are expressed as Hedges’ *g* with 95% confidence intervals. The pooled estimate was obtained using a REML random-effects model with HKSJ adjustment. The pooled Hedges’ *g* was −1.34 (95% HKSJ-adjusted CI [−3.55, +0.86]; *t*(2) = −2.63; *p* = 0.119), with very high between-study heterogeneity (*Q* = 24.69, df = 2, *p* < 0.001; *I^2^* = 90.7%; *τ^2^* = 0.723). The 95% confidence interval crossed the null. **(B)** Leave-one-out analysis. Pooled Hedges’ *g* values after sequential single-study omission were −1.20 (excluding Yu B. et al., 2024), −1.88 (excluding Diao et al., 2025), and −1.00 (excluding Wang et al., 2025). The very wide HKSJ-adjusted confidence intervals under the *k* = 2 leave-one-out iterations reflect the limitations of HKSJ inference at this sample size and should be interpreted as evidence of statistical instability rather than as informative estimates. Negative Hedges’ *g* values indicate lower DTI-ALPS index values in T2DM relative to healthy controls.

Three studies [Yu B 2024; Diao 2025 (T2DM-MCI subgroup); Wang 2025] contributed to the primary analysis of the complicated T2DM subgroup, encompassing 104 patients with T2DM complications and 102 healthy controls. Study-level Hedges’ *g* values were: Yu B 2024, *g* = −1.66; Diao 2025, *g* = −0.37; Wang 2025, *g* = −2.04. All three point estimates were in the direction of lower DTI-ALPS indices in the complicated T2DM groups relative to healthy controls, with Yu B 2024 and Wang 2025 indicating large negative effect sizes and Diao 2025 indicating a small negative effect size (Figure 3A).

Under the REML random-effects model with HKSJ adjustment, the pooled Hedges’ *g* was −1.34 [95% CI (−3.55, +0.86), HKSJ-adjusted; *t*(2) = −2.63; *p* = 0.119]. Between-study heterogeneity was very high (Cochran’s *Q* = 24.69, df = 2, *p* < 0.001; *I^2^* = 90.7%; *τ^2^* = 0.723). The primary analysis of the complicated T2DM subgroup therefore did not reach conventional statistical significance under HKSJ-adjusted inference, and the confidence interval crossed the null. The wide HKSJ-adjusted confidence interval reflects both the very small number of included studies (*k* = 3) and the substantial between-study heterogeneity.

Leave-one-out sensitivity analysis (Figure 3B) indicated that the primary complicated-subgroup pooled estimate was statistically unstable under the small-k conditions of this analysis. Omission of any single study produced HKSJ-adjusted confidence intervals substantially wider than the full *k* = 3 analysis: excluding Yu B 2024 yielded a pooled *g* of −1.20 [95% CI (−11.84, +9.44)]; excluding Wang 2025 yielded a pooled *g* of −1.00 [95% CI (−9.23, +7.24)]; and excluding Diao 2025 yielded a pooled *g* of −1.88 [95% CI (−4.25, +0.49)]. Although the direction of effect was preserved in all leave-one-out iterations, the confidence intervals in the *k* = 2 leave-one-out analyses are too wide to support robust inference. We interpret this finding as evidence that the primary complicated-subgroup analysis is limited not primarily by the magnitude of the effect but by the very small number of eligible studies and the corresponding limitations of HKSJ-adjusted inference under small-k conditions.

### Formal between-subgroup test: primary analysis (*k* = 7)

To directly test whether the two clinical-phenotype subgroups differed in their pooled DTI-ALPS effect estimates, we conducted a formal between-subgroup comparison using Cochran’s *Q*_between_ statistic. In the primary analysis (*k* = 7), the uncomplicated subgroup pooled Hedges’ *g* was −0.89 and the complicated subgroup pooled Hedges’ *g* was −1.34, corresponding to a between-subgroup difference of Δ*g* = −0.45. The formal test yielded *Q*_between_ = 0.66 (df = 1), *p* = 0.416, which did not reach statistical significance.

We therefore conclude that the primary analysis does not provide statistical support for a difference in pooled DTI-ALPS effect sizes between the two clinical-phenotype subgroups. Although the complicated-subgroup point estimate was numerically larger than the uncomplicated-subgroup point estimate, the formal test of between-subgroup difference did not reach the conventional *α* = 0.05 threshold. This result is consistent with, and interpretively cautions, the observation that the two subgroup point estimates differ in magnitude, and it should be considered when interpreting any description of “differences between clinical subgroups” in the present synthesis.

### Sensitivity analysis: complicated T2DM subgroup including Hu 2025

A *post-hoc* sensitivity analysis of the complicated T2DM subgroup incorporated Hu 2025. This study was identified during data extraction as having a substantially smaller control-group standard deviation of the DTI-ALPS index (SD = 0.055) than the other included studies. Because inverse-variance weighting in random-effects meta-analysis is sensitive to reported study precision, this sensitivity analysis was designed to assess whether the inclusion of a high-precision study would alter the direction, magnitude, or statistical stability of the complicated-subgroup pooled estimate.

Consistent with the pre-specified phenotypic classification used throughout the synthesis, the T2DM-MCI subgroup of Hu 2025 (*n* = 35) versus the healthy control group (*n* = 35) was included in the complicated T2DM subgroup. The study-level Hedges’ *g* for this comparison was −2.92 (SE = 0.34), in the same direction as the other three studies in the complicated subgroup.

In the sensitivity analysis (*k* = 4; Supplementary Figure 2A), the pooled Hedges’ *g* under the REML random-effects model with HKSJ adjustment was −1.73 [95% CI (−3.42, −0.04), HKSJ-adjusted; *t*(3) = −3.25; *p* = 0.047], with Cochran’s *Q* = 45.82 (df = 3, *p* < 0.001), *I^2^* = 92.8%, and *τ^2^* = 1.053. The 95% HKSJ-adjusted confidence interval for the sensitivity-analysis pooled estimate marginally excluded the null, corresponding to a nominal *p* value of 0.047. The direction of effect in the sensitivity analysis was consistent with that of the primary analysis, and the magnitude of the pooled estimate was somewhat larger.

Leave-one-out examination of the sensitivity analysis (Supplementary Figure 2B) produced pooled *g* values ranging from −1.34 (omitting Hu 2025) to −2.20 (omitting Diao 2025), with consistent direction of effect across all iterations. Compared with the leave-one-out profile of the primary analysis, the leave-one-out HKSJ confidence intervals in the sensitivity analysis were substantially narrower: for example, omitting Diao 2025 yielded a pooled *g* of −2.20 [95% CI (−3.77, −0.63); *p* = 0.027]. These features indicate that the sensitivity analysis provides a more stable platform for HKSJ-adjusted inference than the primary analysis, consistent with the expectation that HKSJ inference becomes more informative as *k* increases beyond the lower bound of *k* = 3.

We emphasize that the nominal statistical significance observed in the sensitivity analysis should be interpreted with caution. The upper bound of the 95% confidence interval marginally excluded the null, the between-study heterogeneity remained very high (*I*^2^ = 92.8%), and the certainty of evidence across both the primary and sensitivity analyses remains very low under GRADE. The sensitivity-analysis pooled estimate is therefore best interpreted as exploratory evidence supporting the direction of effect observed in the primary analysis, rather than as a confirmatory demonstration of a specific effect size.

### Formal between-subgroup difference test: sensitivity analysis

In the sensitivity analysis including Hu 2025 (*k* = 8 studies across both subgroups), the formal Cochran *Q*_between_ test of the difference between the uncomplicated and complicated subgroup pooled estimates yielded *Q*_between_ = 2.16 (df = 1), *p* = 0.141, which did not reach statistical significance. The between-subgroup difference in the sensitivity analysis was Δ*g* = −0.84, corresponding to the difference between the uncomplicated pooled estimate (*g* = −0.89) and the complicated-with-Hu pooled estimate (*g* = −1.73).

Neither the primary analysis (*p* = 0.416) nor the sensitivity analysis (*p* = 0.141) provided statistical support for a difference between the two clinical-phenotype subgroups. Although the sensitivity-analysis point estimates differed in the expected direction, with a larger magnitude in the complicated subgroup, the formal between-subgroup test did not reach the conventional *α* = 0.05 threshold in either analysis. These results are consistent with the limited statistical resolution of the current evidence base with respect to subgroup-level differences in DTI-ALPS behavior in T2DM, and reinforce the interpretive framing of the present synthesis as exploratory and hypothesis-generating.

### Risk of bias and quality assessment

**Figure 4:**
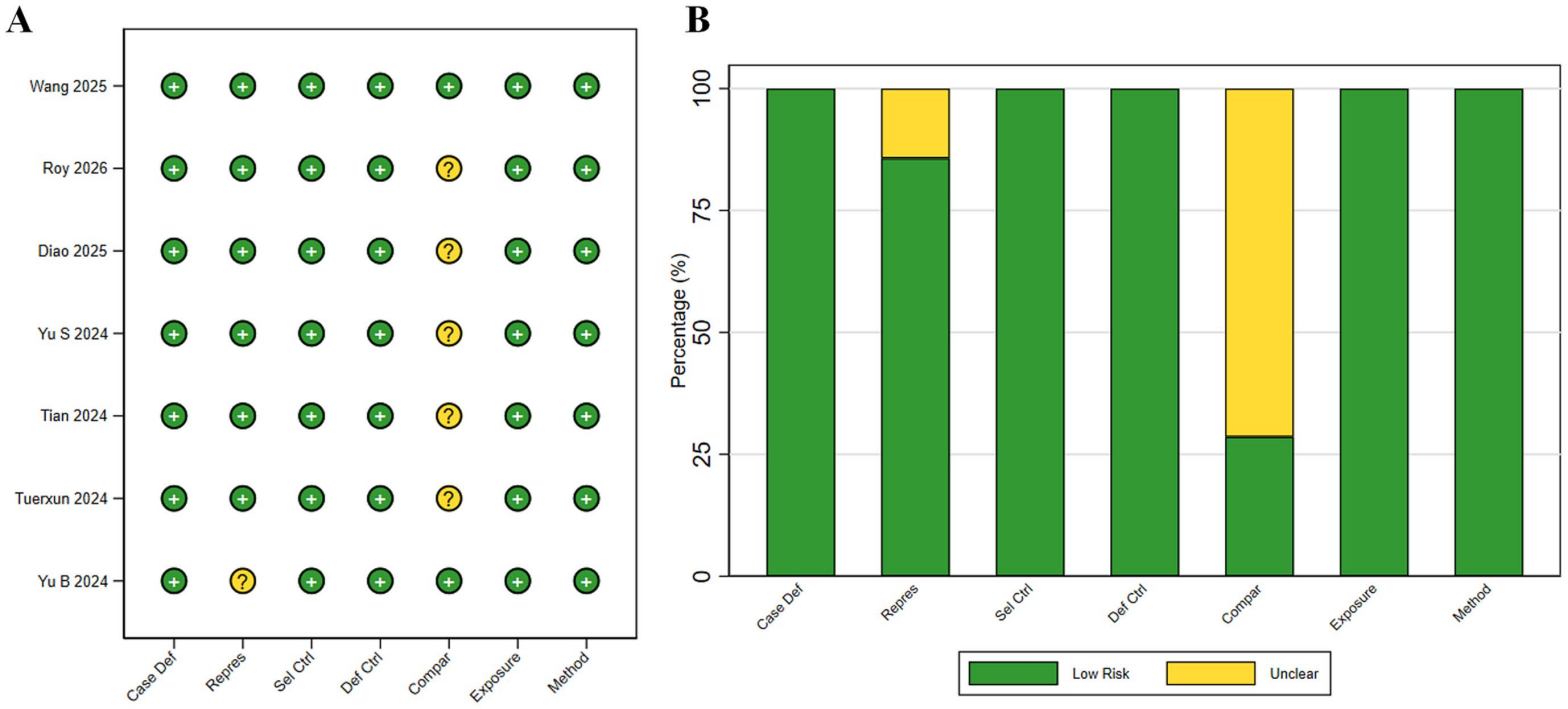
Methodological quality assessment of included studies based on the Newcastle–Ottawa Scale (NOS). **(A)** Item-level summary across the seven NOS domains, with each circle indicating the per-study rating for one domain. **(B)** Proportion of studies rated as low risk or unclear within each domain. NOS domain abbreviations: Case Def, adequacy of case definition; Repres, representativeness of cases; Sel Ctrl, selection of controls; Def Ctrl, definition of controls; Compar, comparability of cases and controls on the basis of the design or analysis; Exposure, ascertainment of exposure; Method, assessment method for outcome ascertainment. Overall, included studies were judged to be of moderate to high methodological quality, with the most common limitation occurring in the comparability domain.

The methodological quality of the included studies was assessed using the Newcastle–Ottawa Scale (NOS). As shown in Supplementary Table 2, NOS scores across the seven studies in the primary synthesis ranged from 7 to 8 stars, indicating overall moderate to high methodological quality. Wang 2025 achieved the highest score of 8 stars. According to the risk-of-bias summary (Figures 4A,B), most studies demonstrated a low risk of bias in the Selection and Exposure domains. The Comparability domain represented the most common methodological limitation, with only 2 of 7 studies (28.6%) in the primary synthesis adjusting for all major confounding factors. These findings indicate that, while the included studies were generally of acceptable methodological quality, residual confounding related to incomplete adjustment should be considered when interpreting the DTI-ALPS–derived findings. Hu 2025, contributing to the sensitivity analysis, received an NOS score of 8 (Supplementary Table 2).

### Certainty of evidence

The certainty of evidence for each primary outcome was assessed using the GRADE framework and is summarized in Supplementary Table 4. For both primary outcomes, the certainty was rated as very low. Evidence from observational studies started at a low level of certainty and was further downgraded based on the following considerations. For the primary outcome of uncomplicated T2DM versus healthy controls, the evidence was downgraded by one level for inconsistency (*I*^2^ = 57.9%) and by one level for imprecision (HKSJ-adjusted 95% confidence interval based on only *k* = 4 contributing studies). For the primary outcome of complicated T2DM versus healthy controls, the evidence was similarly downgraded for inconsistency (*I*^2^ = 90.7%) and imprecision [HKSJ-adjusted 95% CI (−3.55, +0.86), crossing the null; *k* = 3]. The small number of studies and the leave-one-out instability of the complicated primary analysis further support the very low certainty rating.

No downgrade was applied for risk of bias, indirectness, or publication bias in either primary analysis. Despite the very low certainty ratings, the direction of effect was consistent across all seven included studies in the primary synthesis and across all leave-one-out iterations. The very low certainty of evidence, together with the small number of eligible studies, the substantial between-study heterogeneity, and the non-significant formal between-subgroup test in the primary analysis, reinforces the interpretive framing of the present work as an exploratory, hypothesis-generating synthesis rather than as a confirmatory meta-analysis.

## Discussion

### Principal findings

The present meta-analysis synthesizes current evidence on DTI-ALPS–derived perivascular diffusion metrics in T2DM. Across seven eligible studies, pooled point estimates consistently indicated lower DTI-ALPS indices in T2DM than in healthy controls. In the primary analysis, the uncomplicated T2DM subgroup reached statistical significance under HKSJ-adjusted inference [pooled Hedges’ *g* = −0.89, 95% CI (−1.45, −0.34), *p* = 0.015], whereas the complicated T2DM subgroup did not (pooled g = −1.34, *p* = 0.119), reflecting both the very small number of contributing studies in that subgroup (*k* = 3) and the very high heterogeneity (*I*^2^ = 90.7%). In addition, the formal between-subgroup test did not support a statistically significant difference between the two clinical phenotypes (*Q*_between_ = 0.66, df = 1, *p* = 0.416). Even in the *post-hoc* sensitivity analysis, which yielded a marginally significant pooled effect for the complicated subgroup (*p* = 0.047), the between-subgroup contrast remained non-significant (*p* = 0.141). Taken together, these findings provide exploratory evidence consistent with reduced DTI-ALPS indices in T2DM, but they do not provide confirmatory evidence for a specific disease mechanism or for a difference between the two clinical subgroups.

### Methodological considerations regarding DTI-ALPS

The interpretation of the present findings depends critically on framing DTI-ALPS appropriately as an imaging metric. DTI-ALPS is an indirect proxy for perivascular water diffusivity along the direction of medullary veins at the level of the lateral ventricle body, and several methodological considerations warrant explicit acknowledgement.

First, DTI-ALPS has not been directly validated against *in vivo* measurements of interstitial fluid dynamics along glymphatic-related pathways in humans. Although the index has been compared with findings from intrathecal gadolinium-based contrast studies in specific clinical populations, no direct human-validation study has established a one-to-one correspondence between DTI-ALPS values and glymphatic clearance efficiency. DTI-ALPS should therefore be described as a candidate imaging metric rather than as a direct measure of glymphatic function.

Second, the ALPS method does not use susceptibility-weighted imaging, angiography, or any other method to segment out the cerebral vasculature. The claim that DTI-ALPS quantifies perivascular water diffusivity rests on the anatomical assumption that, at the level of the lateral ventricle body, the medullary veins run perpendicular to the projection and association fiber tracts. This anatomical assumption is reasonable but has not been directly verified on a per-subject basis in any of the included studies.

Third, T2DM is independently associated with diffuse alterations in white-matter DTI metrics, including reduced fractional anisotropy and altered mean diffusivity, across multiple brain regions. It is therefore possible that the observed reductions in DTI-ALPS values in T2DM reflect, at least in part, a localized manifestation of these diffuse microstructural changes, rather than a specific impairment of perivascular diffusion dynamics. Reduced DTI-ALPS values in T2DM are therefore most appropriately interpreted as indicators of altered perivascular diffusion behavior, rather than as direct evidence of impaired glymphatic clearance.

These considerations do not invalidate DTI-ALPS as an imaging metric, but they do constrain interpretation of the findings synthesized here. Accordingly, we do not claim that this meta-analysis demonstrates glymphatic dysfunction in T2DM. Rather, the present findings describe the behavior of a candidate imaging metric across a limited number of cohorts and should be interpreted as exploratory and hypothesis-generating.

### Metabolic stress and perivascular vulnerability

The central observation of reduced DTI-ALPS–derived indices in T2DM is consistent with alterations in perivascular diffusion behavior in the setting of chronic metabolic stress. Sustained hyperglycemia promotes the accumulation of advanced glycation end-products (AGEs), which can activate pro-inflammatory signaling cascades and contribute to remodeling of the neurovascular unit (Brownlee, 2005; Hawkins and Davis, 2005). These processes could plausibly disrupt the perivascular microenvironment required for efficient fluid exchange along perivascular spaces (Iliff et al., 2012).

Metabolic and inflammatory stress may also contribute to depolarization of aquaporin-4 (AQP4) water channels on astrocytic endfeet (Kress et al., 2014; Ma and Zhang, 2026). Because polarized AQP4 localization is thought to facilitate cerebrospinal fluid–interstitial fluid exchange, such alterations could plausibly impair perivascular fluid transport (Mestre et al., 2018). In parallel, diabetes-related microvascular stiffening and endothelial dysfunction may attenuate arterial pulsatility, a proposed driving force of perivascular fluid movement, thereby further constraining clearance efficiency (Tarasoff-Conway et al., 2015). Taken together, these mechanisms suggest a plausible link between chronic metabolic disturbance and altered perivascular diffusion behavior in T2DM (see Supplementary Figure 1 for a conceptual overview), but this interpretation remains hypothesis-generating rather than directly demonstrated by the present data.

### Clinical phenotype and cognitive vulnerability

Across studies, DTI-ALPS–derived metrics differed between cohorts with uncomplicated T2DM and those with documented complications. The uncomplicated subgroup point estimate (*g* = −0.89) was numerically smaller in magnitude than the complicated subgroup estimate (*g* = −1.34 in the primary analysis; *g* = −1.73 in the sensitivity analysis), and all seven primary studies reported point estimates in the direction of lower DTI-ALPS indices in T2DM. However, the formal between-subgroup test did not reach statistical significance in either the primary (*p* = 0.416) or sensitivity analysis (*p* = 0.141), and any apparent difference should be interpreted as exploratory evidence warranting replication.

Moreover, the distinction between uncomplicated and complicated T2DM reflects a cohort-level phenotypic classification rather than a temporal stage of disease progression. Cohorts differ not only in complication status but also in age, disease duration, HbA1c, and imaging parameters. The numerically larger effect in the complicated subgroup is therefore consistent with several possible explanations, including greater cumulative metabolic burden, concurrent cognitive impairment contributing to diffusion metrics, or heterogeneity of imaging protocols. The observation that cohorts with manifest cognitive impairment tended to report larger DTI-ALPS reductions is consistent with prior reports that DTI-ALPS metrics covary with cognitive performance across clinical populations (Taoka et al., 2017; Hsiao et al., 2023; Xue et al., 2025), but whether this reflects a causal mechanism or non-specific white-matter injury cannot be determined from cross-sectional data (Rasmussen et al., 2018).

### Technical robustness and methodological consistency

The substantial between-study heterogeneity observed in both primary analyses indicates that variability extends beyond random sampling error. Several sources plausibly contribute to this heterogeneity. At the cohort level, the included studies differ in age, disease duration, HbA1c, BMI, sex distribution, cognitive profile, and the severity of documented complications. Variability in metabolic profiles and treatment exposure across cohorts may further contribute to heterogeneity in effect estimates (van Elderen et al., 2010).

At the acquisition and processing level, the included studies differ in several methodological respects (Table 1). Diffusion-encoding directions varied substantially, ranging from 30 to 99 directions. Higher angular resolution improves tensor estimation stability and may enhance sensitivity to directional diffusion. This could potentially bias ALPS values upward in studies employing extensive diffusion sampling (Jones, 2004; Le Bihan et al., 2001). Furthermore, studies differed in b-value (800 s/mm^2^ in Roy 2026 versus 1,000 s/mm^2^ in the remaining studies), voxel size, post-processing software (FSL vs. DTI Studio), and the reported ALPS index laterality (left, right, or bilateral average).

Crucially, ROI localization strategies also differed. Most studies placed regions of interest in standard space, assuming an anatomical perpendicularity between medullary veins and projection fibers. In contrast, Hu 2025 performed rigid co-registration between DTI and susceptibility-weighted imaging (SWI) to guide the identification of deep medullary veins perpendicular to the lateral ventricle walls and to direct subsequent ROI placement. This methodological refinement plausibly contributes to the higher precision of the control-group DTI-ALPS estimate observed in that study. It provides an anatomical, rather than purely distributional, explanation for the narrow standard deviation reported by Hu 2025.

Formal meta-regression to isolate the effect of individual methodological variables was not performed. The limited number of eligible studies would render such analyses statistically underpowered and potentially misleading. Taken together, the observed heterogeneity reinforces the exploratory framing of the present synthesis and underscores the need for methodologically harmonized future studies to improve comparability across cohorts.

### Strengths and limitations

This study provides a quantitative synthesis of DTI-ALPS–derived metrics in individuals with T2DM, enabling cross-cohort evaluation of perivascular diffusion alterations across pre-specified clinical-phenotype subgroups. By incorporating a formal between-subgroup test, a *post-hoc* sensitivity analysis, leave-one-out diagnostics, and transparent handling of data extraction and methodological decisions, the synthesis provides a structured assessment of how DTI-ALPS–derived metrics behave across a limited but representative sample of the current evidence base.

Several limitations merit explicit consideration. First, the number of eligible studies was small (*k* = 4 in the uncomplicated subgroup and *k* = 3 in the complicated primary analysis), which severely limited the statistical resolution of the synthesis. Under the conservative HKSJ adjustment appropriate for small-k random-effects meta-analysis, the primary analysis of the complicated subgroup did not reach conventional statistical significance, and leave-one-out analysis revealed substantial instability under single-study omission. These features likely reflect the limited current evidence base rather than the absence of a signal.

Second, substantial between-study heterogeneity was observed in both subgroups, reflecting differences in clinical characteristics, imaging protocols, and processing pipelines across cohorts.

Third, although DTI-ALPS is widely used as a non-invasive imaging metric, it remains an indirect proxy for perivascular water diffusivity rather than a direct measure of cerebrospinal fluid–interstitial fluid exchange. Accordingly, reduced DTI-ALPS values in T2DM cannot be interpreted as direct evidence of impaired glymphatic clearance.

Fourth, white matter hyperintensities, which are highly prevalent in T2DM and may influence diffusion metrics in periventricular regions, were not quantified in most included studies, precluding formal adjustment for this potential confounder at the meta-analytic level.

Fifth, the predominance of cross-sectional study designs limits causal inference. The present synthesis cannot determine whether metabolic control modifies perivascular diffusion metrics over time, whether DTI-ALPS changes precede or follow cognitive decline in T2DM, or whether observed differences between clinical subgroups reflect within-individual progression rather than cohort-level differences in composition.

Sixth, the certainty of evidence, assessed using the GRADE framework, was very low for both primary outcomes. This rating was driven by the observational nature of the included studies, between-study heterogeneity, and imprecision associated with the limited number of studies and the wide HKSJ-adjusted confidence intervals of the complicated subgroup analysis. Taken together, these limitations reinforce the exploratory framing of this synthesis and support its interpretation as hypothesis-generating rather than confirmatory.

Seventh, one study required graphical extraction and conversion of summary statistics, which introduces greater measurement uncertainty than direct tabular extraction despite preservation of effect direction in leave-one-out analysis.

## Conclusion

This exploratory meta-analysis synthesizes the current evidence on DTI-ALPS perivascular diffusion metrics in type 2 diabetes mellitus. Across 532 participants, point estimates consistently trended toward lower DTI-ALPS indices in T2DM compared with healthy controls. The uncomplicated T2DM subgroup primary analysis reached statistical significance under HKSJ-adjusted inference [pooled Hedges’ *g* = −0.89, 95% CI (−1.45, −0.34), *p* = 0.015], whereas the complicated T2DM subgroup primary analysis did not (*p* = 0.119), reflecting the very small number of contributing studies and very high heterogeneity in that subgroup. Formal testing did not support a definitive difference between uncomplicated and complicated clinical phenotypes. Several major limitations characterize the current evidence base, including the small number of eligible studies, substantial between-study heterogeneity in the complicated subgroup, instability of the complicated-subgroup analysis under leave-one-out examination, the indirect nature of DTI-ALPS as a proxy for perivascular fluid dynamics, and potential confounding by white-matter microangiopathy in T2DM. Consequently, the available evidence justifies further investigation of DTI-ALPS as a candidate imaging metric, but it is insufficient to support definitive claims regarding glymphatic dysfunction in diabetes. Future research requires large, prospectively designed, and methodologically homogeneous cohorts. Incorporating longitudinal follow-up, harmonized image processing pipelines, and cross-validation against independent fluid dynamics measures will be essential to determine the utility of DTI-ALPS in characterizing metabolic brain vulnerability.

## Data Availability

The datasets presented in this study can be found in online repositories. The names of the repository/repositories and accession number(s) can be found in the article/Supplementary materials.
