# Supplementary material for: Glymphatic-related imaging findings in type 2 diabetes mellitus: a systematic review and exploratory meta-analysis of DTI-ALPS studies
Source: Front Aging Neurosci. 2026 May 21;18:1818446. doi: 10.3389/fnagi.2026.1818446 (PMC13233433; doi:10.3389/fnagi.2026.1818446)
Supplement: Supplementary file 1 [file Data_Sheet_1.PDF]

Supplementary Methods

This supplement provides extraction procedures, source-paragraph references, and decision rationales for individual studies whose data extraction required individualized handling, together with additional methodological details summarized but not fully described in the main Methods.

S1. Yu B 2024: Graphical extraction from violin plots

The original publication of Yu B 2024 (Yu et al., 2024) did not report tabular mean ± standard deviation values for the DTI-ALPS index. Group-level distributions were instead displayed as violin plots in Figure 3, with overlaid dashed markers indicating the median and the first and third quartiles (Q_1 and Q_3) of each group. We read the median, Q_1, and Q_3 values directly from these markers and converted them to mean ± SD using the Wan et al. (2014) formulas, consistent with the median–IQR conversion procedure described in the main Methods. The resulting values were: DKD group, mean = 1.18, SD = 0.15 (n = 25); healthy control group, mean = 1.46, SD = 0.18 (n = 25). This approach differs from graphical reconstruction of individual data points from marker-free scatter plots, which was the basis for excluding Yang 2020 (see **Section S4**). Because violin plots display the median and quartile values directly as figure elements, the Wan conversion (Wan et al., 2014) is appropriate for this category of input. The influence of Yu B 2024 on the pooled complicated-subgroup estimate is evaluated in the pre-specified leave-one-out analysis (main **Figure 3B**).

S2. Tian 2024: Reconstruction from published 95% confidence intervals

The original publication of Tian 2024 (Tian et al., 2024) did not report tabular mean ± standard deviation values for the DTI-ALPS index. Group-level summary statistics were reported in the Results text as 95% confidence intervals and visualized as box plots in **Figure 2**.

S2.1 Reconstruction formula

Group means and standard deviations were reconstructed by inverting the t-distribution-based 95% CI formula, in accordance with Cochrane Handbook Chapter 6.5.2.3 (Higgins et al., 2023). For a confidence interval (lower, upper) reported for a sample of size n: (For n = 22, $t_{0.975,\,21}$ = 2.0796).

$$\text{Mean} = (\text{lower} + \text{upper}) / 2$$

$$SD = (\text{upper} - \text{lower}) \times \sqrt{n} / (2 \times t_{0.975,\, n-1})$$

S2.2 Identification of the source CI for each group

The Results text of Tian 2024 reports two pairwise comparisons in two consecutive paragraphs (T2DM vs. NGM in the first; T2DM vs. prediabetes in the second), and the same numerical confidence interval (1.672 to 1.909) appears in both. Identification of the correct CI for each group therefore required cross-checking within the publication.

The second paragraph reports T2DM vs. prediabetes as "95%CI: 1.461 to 1.687 vs 1.672 to 1.909," with the T2DM group described as significantly reduced ($P = 0.010$). This unambiguously identifies 1.461 to 1.687 as the T2DM CI and 1.672 to 1.909 as the prediabetes CI. The assignment is corroborated by the box plot in Figure 2C, which visually places the T2DM group median at approximately 1.5 and the NGM and prediabetes medians at approximately 1.8.

The first paragraph reports T2DM vs. NGM as "95%CI: 1.672 to 1.909 vs 1.722 to 1.949." Under the cross-validated reading from the second paragraph, the value 1.672 to 1.909 in this position must correspond to the prediabetes group rather than to T2DM, leaving 1.722 to 1.949 as the NGM CI. The face-value reading of the first paragraph alone is internally inconsistent with both the second paragraph and Figure 2C, and was therefore not adopted as the primary extraction.

S2.3 Resulting summary statistics

Applying the formula in S2.1 to the CIs identified in S2.2 yielded:

- T2DM group (CI 1.461 to 1.687, $n = 22$): mean = 1.5740, SD = 0.2549
- Healthy control (NGM) group (CI 1.722 to 1.949, $n = 22$): mean = 1.8355, SD = 0.2560

S2.4 Pre-specified sensitivity analysis using an alternative reading

We also conducted a pre-specified sensitivity analysis using the face-value reading of the first paragraph, under which T2DM is assigned mean = 1.7905, SD = 0.2673 and NGM remains mean = 1.8355, SD = 0.2560. Under this alternative extraction, the uncomplicated T2DM subgroup pooled Hedges' g was −0.73 (95% HKSJ-adjusted CI [−1.50, +0.05], $p = 0.06$), preserving the direction of effect but not reaching conventional statistical significance; the leave-

one-out range was −0.60 to −0.88. Complete results, including a side-by-side comparison with the primary extraction, are reported in Section S6.

S3. Diao 2025: Multi-arm study handling

Diao 2025 (Diao et al., 2025) is a three-arm study reporting healthy controls (n = 37), T2DM without cognitive impairment (n = 37), and T2DM with mild cognitive impairment (n = 39). For the present synthesis we included a single comparison: the T2DM-MCI subgroup versus the healthy control group, classified in the "T2DM with complications" subgroup in accordance with our pre-specified phenotypic classification. The T2DM without MCI subgroup was not included, to avoid the unit-of-analysis concerns that would arise from using the same control group in more than one comparison within a single meta-analysis (Higgins et al., 2023, Chapter 6.2). Because the DTI-ALPS index in Diao 2025 was reported as median (interquartile range), summary statistics were converted using the Wan et al. (2014) formulas. The resulting values were: T2DM-MCI, mean = 1.60, SD = 0.20 (n = 39); healthy control, mean = 1.68, SD = 0.23 (n = 37). The same unit-of-analysis principle was applied to Hu 2025 in the post-hoc sensitivity analysis: only the T2DM-MCI subgroup (n = 35) versus the healthy control group (n = 35) was used.

S4. Yang 2020 and Xu 2026: Detailed exclusion rationales

S4.1 Yang 2020

Yang 2020 (Yang et al., 2020) met the population, intervention, and outcome eligibility criteria, but the original publication presented DTI-ALPS values only as scatter plots without overlaid summary markers (no group means, medians, error bars, or interquartile range markers from which group-level summary statistics could be reliably read). Direct extraction of mean ± SD or median–IQR was therefore not possible. Reconstruction of summary statistics by graphical reading of individual data points from a marker-free scatter plot was considered but was deemed insufficiently reliable: individual-point reconstruction is sensitive to point-overlap and image-resolution artifacts, and is methodologically distinct from the graphical median–IQR reading applied to Yu B 2024 (Section S1), in which the summary markers themselves were displayed as discrete figure elements. Yang 2020 was therefore not included in the primary quantitative synthesis.

S4.2 Xu 2026

Xu 2026 (Xu et al., 2026) addressed the relevant population and outcome but did not report sufficient quantitative data to permit valid effect-size calculation. The summary statistics required for Hedges' *g* computation (group means, standard deviations, and sample sizes for both T2DM and healthy control groups) were not reported in a directly extractable form, and no alternative summary measures (standard errors, *t* statistics, or *F* statistics with degrees of freedom) from which standard deviations could be reconstructed were provided. Xu 2026 was therefore not included.

S5. MRI acquisition and processing: common elements and sources of variability

The following acquisition and processing elements were common to all included studies: 1) 3.0 Tesla magnetic field strength. 2) Single-shot echo-planar diffusion-weighted imaging. 3) Bilateral region-of-interest placement at the level of the lateral ventricle body, in areas corresponding to projection fibers (medial) and association fibers (lateral). 4) Calculation of the DTI-ALPS index using the formula proposed by Taoka (Taoka et al., 2017). The following elements varied across studies; per-study values are tabulated in main Table 1:

Element	Range / categories observed
b-value	800 s/mm² (Roy 2026); 1000 s/mm² (all other studies)
Diffusion-encoding directions	30 to 99
Post-processing software	FSL (five studies); DTI Studio (two studies)
Voxel size	1.7 × 1.7 × 1.7 mm³ to 3.0 × 2.0 × 2.0 mm³
ROI placement strategy	Manual or template-based (MNI-registered)
Reported ALPS index laterality	Left only; right only; bilateral average

The standard pre-processing pipeline used across studies consisted of: (1) correction for eddy-current distortions, susceptibility-induced distortions, and head motion using FSL (Jenkinson et al., 2012) or equivalent toolboxes, with the FSL "eddy" tool employed for geometric distortion correction (Andersson and Sotiropoulos, 2016); (2) voxel-wise diffusion tensor estimation; (3) extraction of diffusivity values along the x-axis in the projection fiber

region ($D_{xx,proj}$) and association fiber region ($D_{xx,assoc}$), together with the perpendicular diffusivities ($D_{yy,proj}$ and $D_{zz,assoc}$); and (4) computation of the DTI-ALPS index as:

$$\text{ALPS index} = \text{mean}(D_{xx,proj}, D_{xx,assoc}) / \text{mean}(D_{yy,proj}, D_{zz,assoc})$$

These methodological differences are discussed in the main Discussion as plausible contributors to between-study heterogeneity. Because of the limited number of eligible studies, formal meta-regression to isolate the effect of individual variables was not performed.

S6. Sensitivity analysis: alternative extraction of Tian 2024

This section reports the complete results of the pre-specified sensitivity analysis using the alternative face-value reading of Tian 2024 described in Section S2.4. Under this extraction, the T2DM group of Tian 2024 was assigned mean = 1.7905, SD = 0.2673 (from CI 1.672 to 1.909), and the NGM group was assigned mean = 1.8355, SD = 0.2560 (from CI 1.722 to 1.949). All other studies retained their primary-extraction values.

S6.1 Side-by-side comparison of primary and alternative extractions

**Analysis**	**Primary extraction (S2.3)**	**Alternative extraction (S2.4)**
Tian 2024 study-level Hedges' *g*	−1.01 [−1.62, −0.39]	−0.17 [−0.75, +0.41]
Uncomplicated subgroup pooled *g* ($k = 4$)	−0.89 [−1.45, −0.34]	−0.73 [−1.50, +0.05]
Uncomplicated subgroup *p* value	0.015	0.06
Uncomplicated subgroup I^2	57.9%	72.6%
Uncomplicated subgroup τ^2	0.090	0.159
Cochran's *Q* (df = 3)	7.74	10.09
Cochran's *Q p* value	0.052	0.018
Leave-one-out range, uncomplicated	−0.82 to −1.11	−0.60 to −0.88
Direction of effect preserved	✓	✓
$k = 7$ $Q_{between}$ (df = 1)	0.66	1.17
$k = 7$ $Q_{between}$ *p* value	0.416	0.279

Analysis	Primary extraction (S2.3)	Alternative extraction (S2.4)
$k = 8$ Q_{between} (df = 1)	2.16	2.94
$k = 8$ Q_{between} p value	0.141	0.086
Complicated subgroup analyses (both $k = 3$ and $k = 4$ with Hu 2025)	Unchanged	Unchanged

S6.2 Interpretation

The direction of effect in the uncomplicated T2DM subgroup is robust to the choice of extraction approach. The qualitative conclusion regarding statistical significance under HKSJ-adjusted inference, however, depends on the extraction approach: the primary extraction yields $p = 0.015$ (significant), whereas the alternative extraction yields $p = 0.06$ (not significant). In both cases, the magnitude of the pooled effect is consistent with a moderate-to-large reduction in DTI-ALPS index in T2DM relative to healthy controls. The complicated T2DM subgroup analyses are unaffected by the choice of Tian 2024 extraction, because Tian 2024 is classified in the uncomplicated subgroup.

S6.3 Reproducibility

Stata reproducibility scripts for both extractions are deposited in the reproducibility package. The two scripts use identical analysis specifications (REML between-study variance estimation, HKSJ small-sample inference, and identical leave-one-out procedures); the only difference is the input mean and SD values for Tian 2024.

Reference

ANDERSSON, J. L. R. & SOTIROPOULOS, S. N. 2016. An integrated approach to correction for off-resonance effects and subject movement in diffusion MR imaging. *Neuroimage,* 125**,** 1063-1078.

DIAO, Z., HUANG, X., SHEN, D., WANG, K., WANG, J., ZHAO, K., ZHAO, C., CAO, Z., TAN, X. & QIU, S. 2025. Analysis of ALPS-Index: Difference in Type 2 Diabetes Mellitus With or Without Mild Cognitive Impairment and Its Relationship With Hippocampal Microstructure. *Brain Behav,* 15**,** e70672.

JENKINSON, M., BECKMANN, C. F., BEHRENS, T. E., WOOLRICH, M. W. & SMITH, S. M. 2012. FSL. *Neuroimage,* 62**,** 782-90.

TAOKA, T., MASUTANI, Y., KAWAI, H., NAKANE, T., MATSUOKA, K., YASUNO, F., KISHIMOTO, T. & NAGANAWA, S. 2017. Evaluation of glymphatic system activity with the diffusion MR technique: diffusion tensor image analysis along the perivascular space (DTI-ALPS) in Alzheimer's disease cases. *Jpn J Radiol,* 35**,** 172-178.

TIAN, B., ZHAO, C., LIANG, J. L., ZHANG, H. T., XU, Y. F., ZHENG, H. L., ZHOU, J., GONG, J. N., LU, S. T. & ZENG, Z. S. 2024. Glymphatic function and its influencing factors in different glucose metabolism states. *WORLD JOURNAL OF DIABETES,* 15.

WAN, X., WANG, W., LIU, J. & TONG, T. 2014. Estimating the sample mean and standard deviation from the sample size, median, range and/or interquartile range. *BMC Med Res Methodol,* 14**,** 135.

XU, S., WANG, X. Y., YANG, D., ZHANG, D., DU, L. N., CHEN, Q. Q., YANG, Y., LIN, L., SHEN, J. & WU, J. L. 2026. Exploring the relationship between lymphatic system damage and insulin resistance in T2DM based on vascular periadventitial tensor analysis and triglyceride-glucose index. *BMC Endocr Disord,* 26**,** 38.

YANG, G., DENG, N., LIU, Y., GU, Y. & YAO, X. 2020. Evaluation of Glymphatic System Using Diffusion MR Technique in T2DM Cases. *Front Hum Neurosci,* 14**,** 300.

YU, B., YANG, W., SONG, L., LI, M., XU, B., WANG, Z., DIAO, Z. & WANG, H. 2024. Glymphatic system dysfunction in nondialysis-dependent ESRD patients with diabetic kidney disease: associations with clinical characteristics and cognitive function. *Ren Fail,* 46**,** 2425160.
