# Supplementary material for: Glymphatic-related imaging findings in type 2 diabetes mellitus: a systematic review and exploratory meta-analysis of DTI-ALPS studies
Source: Front Aging Neurosci. 2026 May 21;18:1818446. doi: 10.3389/fnagi.2026.1818446 (PMC13233433; doi:10.3389/fnagi.2026.1818446)
Supplement: Supplementary file 2 [file Data_Sheet_2.PDF]

Supplementary Table 1. Detailed search strategies for PubMed, Embase, and Web of Science. (Search date update 2026.2.10)

Panel A: PubMed

Step	Search Query	Results
**#1**	"Diabetes Mellitus"[MeSH] OR "Metabolic Syndrome"[MeSH] OR "Insulin Resistance"[MeSH] OR "Diabetes"[TIAB] OR "Metabolic Syndrome"[TIAB] OR "Insulin Resistance"[TIAB]	1,023,359
**#2**	"Glymphatic System"[MeSH] OR "Glymphatic"[TIAB] OR "Waste clearance"[TIAB] OR "Perivascular space"[TIAB] OR "EPVS"[TIAB]	4,511
**#3**	"Diffusion Tensor Imaging"[MeSH] OR "DTI-ALPS"[TIAB] OR "ALPS index"[TIAB]	16,391
**#4**	#1 AND #2 AND #3	26

Panel B: Embase

Step	Search Query	Results
**#1**	'diabetes mellitus'/exp OR 'metabolic syndrome x'/exp OR 'insulin resistance'/exp OR 'diabetes':ti,ab,kw OR 'metabolic syndrome':ti,ab,kw OR 'insulin resistance':ti,ab,kw	1,915,396
**#2**	'glymphatic system'/exp OR 'glymphatic':ti,ab,kw OR 'waste clearance':ti,ab,kw OR 'perivascular space':ti,ab,kw OR 'epvs':ti,ab,kw	6,271
**#3**	'diffusion tensor imaging'/exp OR 'dti-alps':ti,ab,kw OR 'alps index':ti,ab,kw	40,075
**#4**	#1 AND #2 AND #3	65

Panel C: Web of Science

Step	Search Query	Results
**#1**	"Diabetes Mellitus" OR "Type 2 Diabetes" OR "T2DM" OR "Metabolic Syndrome" OR "Insulin Resistance"	707,442
**#2**	"Glymphatic System" OR "Glymphatic" OR "Waste clearance" OR	4,639

	"Perivascular space" OR "EPVS"	
**#3**	"DTI-ALPS" OR "ALPS index" OR "Analysis along the perivascular space"	596
**#4**	#1 AND #2 AND #3	19

Supplementary Table 2. Detailed quality assessment of included studies using the Newcastle-Ottawa Scale (NOS).

Study	Selection (max 4★)	Comparability (max 2★)	Outcome (max 3★)	Total
Wang et al. 2025	★★★★	★★	★★	8
Roy et al. 2026	★★★★	★	★★	7
Diao et al. 2025	★★★★	★	★★	7
Yu S et al. 2024	★★★★	★	★★	7
Tian et al. 2024	★★★★	★	★★	7
Tuerxun et al. 2024	★★★★	★	★★	7
Yu B et al. 2024	★★★	★★	★★	7
Hu et al. 2025	★★★★	★★	★★	8

Although NOS scores ranged from 7 to 8, limitations in the comparability domain were common, and overall study quality should be interpreted as moderate to high rather than uniformly high.

Supplementary Table 3. Sensitivity analysis of the primary meta-analysis using Hartung–Knapp–Sidik–Jonkman (HKSJ)–adjusted random-effects models

Omitted study	k	Hedges' g	95% HKSJ CI	p
**Panel A. Uncomplicated T2DM (k = 4)**				
None (full analysis)	4	−0.89	[−1.45, −0.34]	0.015
Tian et al. 2024	3	−0.88	[−1.90, +0.13]	0.064
Tuerxun et al. 2024	3	−0.82	[−1.71, +0.07]	0.058
Yu S et al. 2024	3	−0.83	[−1.78, +0.13]	0.065
Roy et al. 2026	3	−1.11	[−1.34, −0.88]	0.002
**Panel B. Complicated T2DM — primary analysis (k = 3)**				
None (full analysis)	3	−1.34	[−3.55, +0.86]	0.119
Yu B et al. 2024	2	−1.20	[−11.84, +9.44]	0.388
Diao et al. 2025	2	−1.88	[−4.25, +0.49]	0.063

Wang et al. 2025	2	−1.00	[−9.23, +7.24]	0.367
**Panel C. Complicated T2DM — sensitivity analysis including Hu et al. 2025 (k = 4)**				
None (full analysis)	4	−1.73	[−3.42, −0.04]	0.047
Yu B et al. 2024	3	−1.76	[−4.98, +1.46]	0.143
Diao et al. 2025	3	−2.20	[−3.77, −0.63]	0.027
Wang et al. 2025	3	−1.63	[−4.81, +1.54]	0.157
Hu et al. 2025	3	−1.34	[−3.55, +0.86]	0.119

Abbreviations: *k*, number of studies; HKSJ, Hartung–Knapp–Sidik–Jonkman adjustment; CI, confidence interval. Negative Hedges' g values indicate lower DTI-ALPS index values in T2DM relative to healthy controls. The full-analysis row of Panel C, omit-Hu row of Panel C, and the full-analysis row of Panel B yield identical pooled estimates by construction (omitting Hu et al. 2025 from the $k = 4$ sensitivity set returns the $k = 3$ primary set).

Supplementary Table 4. GRADE summary of findings

Outcome	No. of studies	Participants (T2DM / Controls)	Effect	Certainty of evidence (GRADE)	Reasons for downgrading
DTI-ALPS (uncomplicated T2DM)	4	159/185	g = −0.89, 95% CI [−1.45, -0.34], p = 0.015	●○○○ **Very low**	Inconsistency, Imprecision
DTI-ALPS (T2DM with complications)	3	104/102	g = −1.34, 95% CI [−3.55, +0.86], p = 0.119	●○○○ **Very Low**	Inconsistency, Imprecision

Abbreviations: HC, healthy controls; CI, confidence interval; HKSJ, Hartung–Knapp–Sidik–Jonkman adjustment; GRADE, Grading of Recommendations, Assessment, Development and Evaluations.
Notes: Evidence from observational (cross-sectional) studies starts at "low" certainty in the GRADE framework. No downgrade was applied for risk of bias (NOS 7–8 across all studies), indirectness (populations and outcomes directly relevant), or publication bias (too few studies for formal assessment; no direct evidence identified). No upgrading factors were applied. Certainty ratings: ⊕ ⊕ ⊕ ⊕ High; ⊕ ⊕ ⊕ ○ Moderate; ⊕ ⊕ ○○ Low; ⊕ ○○○ Very low.

Supplementary Figure 1. Conceptual model of metabolic–perivascular dysfunction in T2DM

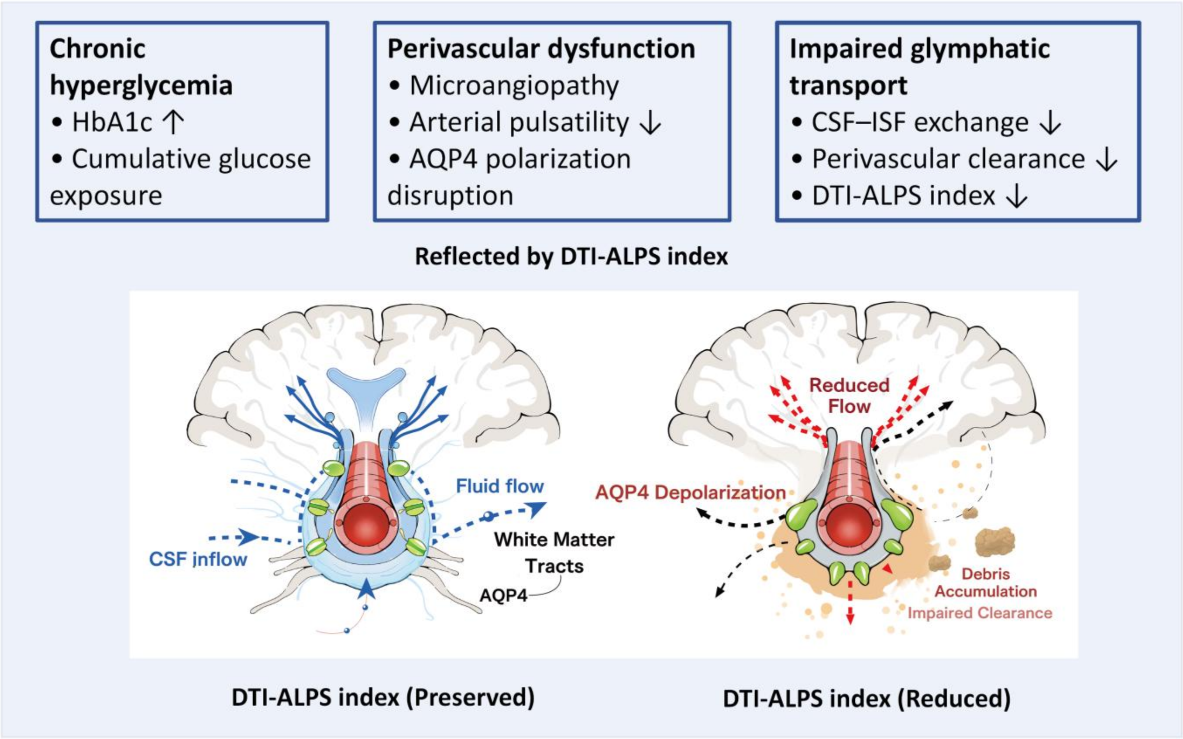

Chronic metabolic stress in T2DM, characterized by sustained hyperglycemia and insulin resistance, is associated with oxidative stress, inflammation, and neurovascular unit remodeling. These processes may influence astrocytic aquaporin-4 (AQP4) polarization and arterial pulsatility, potentially altering perivascular fluid dynamics. Changes in perivascular diffusion patterns may emerge early in the disease course and become more pronounced along the cognitive continuum, with possible implications for the clearance of neurotoxic metabolites such as amyloid-β and tau. This schematic illustrates a hypothesized metabolic–perivascular–cognitive axis based on converging experimental and imaging evidence.

Supplementary Figure 2. Post-hoc sensitivity analysis (complicated T2DM including Hu et al. 2025)

A

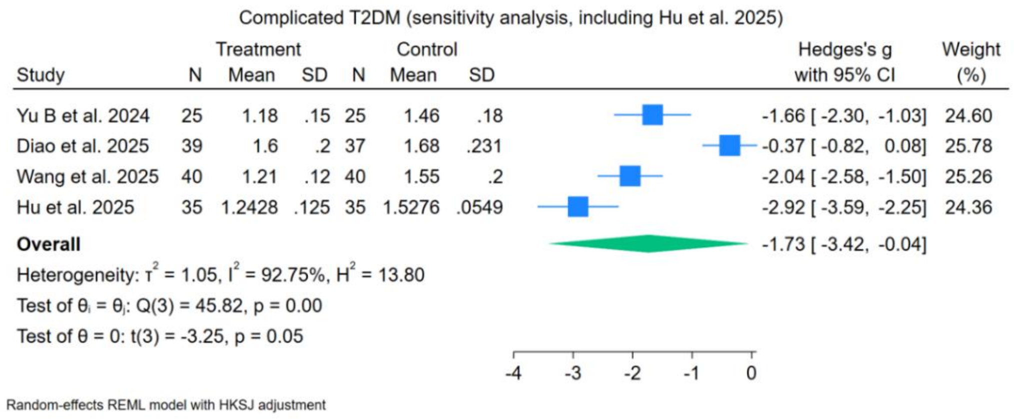

B

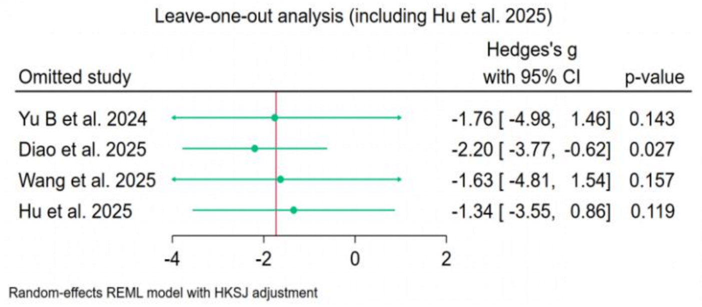

(A) Forest plot of the four studies contributing to the sensitivity analysis. Individual study effect sizes are expressed as Hedges' g with 95% confidence intervals. The pooled estimate was obtained using a REML random-effects model with HKSJ adjustment. The pooled Hedges' g was −1.73 (95% HKSJ-adjusted CI [−3.42, −0.04]; t(3) = −3.25; $p = 0.047$), with very high between-study heterogeneity ($Q = 45.82$, df = 3, $p < 0.001$; $I^2 = 92.8\%$; $\tau^2 = 1.053$). The 95% HKSJ-adjusted confidence interval marginally excluded the null. (B) Leave-one-out analysis. Pooled Hedges' g values after sequential single-study omission were −1.76 (excluding Yu B et al. 2024), −2.20 (excluding Diao et al. 2025), −1.63 (excluding Wang et al. 2025), and −1.34 (excluding Hu et al. 2025). The direction of effect was preserved across all iterations. Negative Hedges' g values indicate lower DTI-ALPS index values in T2DM relative to healthy controls.
